# *PRICKLE1* × *FOCAD* Interaction Revealed by Genome-Wide vQTL Analysis of Human Facial Traits

**DOI:** 10.3389/fgene.2021.674642

**Published:** 2021-08-09

**Authors:** Dongjing Liu, Hyo-Jeong Ban, Ahmed M. El Sergani, Myoung Keun Lee, Jacqueline T. Hecht, George L. Wehby, Lina M. Moreno, Eleanor Feingold, Mary L. Marazita, Seongwon Cha, Heather L. Szabo-Rogers, Seth M. Weinberg, John R. Shaffer

**Affiliations:** ^1^Department of Genetics and Genomic Sciences, Icahn School of Medicine at Mount Sinai, New York, NY, United States; ^2^Future Medicine Division, Korea Institute of Oriental Medicine, Daejeon, South Korea; ^3^Center for Craniofacial and Dental Genetics, School of Dental Medicine, University of Pittsburgh, Pittsburgh, PA, United States; ^4^Department of Oral and Craniofacial Sciences, School of Dental Medicine, University of Pittsburgh, Pittsburgh, PA, United States; ^5^Department of Pediatrics, McGovern Medical Center, The University of Texas Health Science Center at Houston, Houston, TX, United States; ^6^Department of Health Management and Policy, The University of Iowa, Iowa City, IA, United States; ^7^Department of Orthodontics, The University of Iowa, Iowa City, IA, United States; ^8^Department of Human Genetics, Graduate School of Public Health, University of Pittsburgh, Pittsburgh, PA, United States; ^9^Department of Biostatistics, Graduate School of Public Health, University of Pittsburgh, Pittsburgh, PA, United States; ^10^Department of Psychiatry, Clinical and Translational Science Institute, School of Medicine, University of Pittsburgh, Pittsburgh, PA, United States; ^11^Department of Developmental Biology, School of Medicine, University of Pittsburgh, Pittsburgh, PA, United States; ^12^Regenerative Medicine at the McGowan Institute, University of Pittsburgh, Pittsburgh, PA, United States; ^13^Center for Craniofacial Regeneration, School of Dental Medicine, University of Pittsburgh, Pittsburgh, PA, United States

**Keywords:** human facial traits, variance quantitative trait loci (vQTL), gene-by-gene (G × G) interaction, *Prickle1*, Focadhesin, craniofacial

## Abstract

The human face is a highly complex and variable structure resulting from the intricate coordination of numerous genetic and non-genetic factors. Hundreds of genomic loci impacting quantitative facial features have been identified. While these associations have been shown to influence morphology by altering the mean size and shape of facial measures, their effect on trait variance remains unclear. We conducted a genome-wide association analysis for the variance of 20 quantitative facial measurements in 2,447 European individuals and identified several suggestive variance quantitative trait loci (vQTLs). These vQTLs guided us to conduct an efficient search for gene-by-gene (G × G) interactions, which uncovered an interaction between *PRICKLE1* and *FOCAD* affecting cranial base width. We replicated this G × G interaction signal at the locus level in an additional 5,128 Korean individuals. We used the hypomorphic *Prickle1*^*Beetlejuice*^ (*Prickle1*^*Bj*^) mouse line to directly test the function of Prickle1 on the cranial base and observed wider cranial bases in *Prickle1^*Bj/Bj*^.* Importantly, we observed that the Prickle1 and Focadhesin proteins co-localize in murine cranial base chondrocytes, and this co-localization is abnormal in the *Prickle1*^*Bj/Bj*^ mutants. Taken together, our findings uncovered a novel G × G interaction effect in humans with strong support from both epidemiological and molecular studies. These results highlight the potential of studying measures of phenotypic variability in gene mapping studies of facial morphology.

## Introduction

Human genetic studies have been remarkably successful at detecting variants with an impact on mean trait values. On the other hand, the genetic basis of phenotypic variability remains largely uncharacterized. Variance quantitative trait loci (vQTLs) are genetic variants exhibiting inter-individual intra-genotypic variability, where one of the alleles is associated with a larger phenotypic variance compared to the other (Rönnegård and Valdar, 2012). vQTLs are seldom examined in conventional genome-wide association studies (GWASes), which usually assume variance homogeneity across genotype groups and aim to detect differences in group means. Despite this, knowledge of vQTLs may lead to a deeper understanding of genotype–phenotype relationships by providing insights into the genetic control of phenotypic variance and revealing possible interactions among genetic variants.

The human face is a highly complex structure resulting from the intricate coordination of multiple genetic and epigenetic factors. Beyond the individual effect of common single nucleotide polymorphisms (SNPs) identified through GWAS (Liu et al., 2012; Paternoster et al., 2012; Adhikari et al., 2016; Cole et al., 2016; Shaffer et al., 2016; Lee et al., 2017; Cha et al., 2018; Claes et al., 2018; Crouch et al., 2018; White et al., 2020), gene-by-gene (G × G) interactions are also expected to play an essential role. Statistical interaction is typically defined as the deviation from additivity in a linear model (Moore and Williams, 2009). The biological interpretation of a statistical interaction is often a challenging task and requires careful experiments to demonstrate a real crosstalk between the genetic factors in the relevant context (Moore, 2005; Moore and Williams, 2005). Although recognized as one of the critical potential sources of missing heritability (Zuk et al., 2012), G × G interactions remain largely uncharacterized in most human traits due to low statistical power, a high computational burden, and difficulties in uncovering the biological relevance for statistical interactions. A recent study of facial morphology reported some evidence of the potentially coordinated actions of variants on facial variation (White et al., 2020). We now know that facial morphology is influenced by variants at over 200 loci (White et al., 2020), which, in combination, give rise to a vast and usually unknowable interaction map. As a genome-wide search for G × G interactions would be intractable, narrowing the focus to a subset of candidate SNPs is necessary, and effective strategies are needed to guide this pre-selection process.

One sensible strategy is variance prioritization. It has been shown, both theoretically and empirically, that a heterogeneous phenotypic variance across genotype groups can arise from genetic interaction effects (Struchalin et al., 2010; Forsberg and Carlborg, 2017). Although these two non-additive genetic inheritance patterns are not always related, their relationship suggests an enrichment of the interaction effects among variance-controlling SNPs at vQTLs, indicating that these SNPs can be prioritized for interaction tests. Indeed, this variance prioritization strategy has been shown to be useful in studying other quantitative human phenotypes (Paré et al., 2010; Hulse and Cai, 2013; Rask-Andersen et al., 2017; Sarkar et al., 2019; Wang et al., 2019; Young et al., 2018; Johnson et al., 2020; Marderstein et al., 2021).

The aim of this study was twofold: to characterize the genetic control of facial trait variability and to investigate G × G interaction effects by leveraging the identified vQTLs. We conducted genome-wide vQTL analyses for 20 facial measurements in 2,447 individuals. Among the suggestive vQTLs, one was located within *PRICKLE1*, a gene known to contribute to the control of craniofacial development in mouse mutants (Yang et al., 2013; Liu et al., 2014; Gibbs et al., 2016; Wan et al., 2018). We followed up on this vQTL by testing whether it interacts with other loci across the genome. We discovered a novel G × G interaction effect between *PRICKLE1* and *FOCAD* impacting cranial base width, which was statistically replicated in an independent cohort and experimentally verified by showing the co-expression of both genes during the critical stages of mouse craniofacial development. Our findings highlight the importance of studying facial variability in addition to facial mean differences for gene discovery and mechanistic exploration.

## Materials and Methods

### Discovery Cohort

The cohort, facial phenotyping, and SNP genotyping were described in a previous GWAS (Shaffer et al., 2016). In brief, three-dimensional (3D) facial images were collected using digital stereophotogrammetry on a cohort consisting of 2,447 unrelated white individuals of European ancestry from the United States. The cohort ranged in age from 3 to 49 years, had a female proportion of 62%, and was free of any conditions known to affect the face or head. Facial landmarks were manually registered on the surface images, from which we calculated 20 linear distances (Supplementary Figure 1 and Supplementary Table 1). The cohort was genotyped on the Illumina OmniExpress + Exome v1.2 array (Illumina, San Diego, CA, United States) and was then fully imputed to the 1,000 Genomes Project phase 3 reference panel. Ancestry principal components (PCs) based on linkage disequilibrium (LD)-pruned autosomal SNPs were constructed and used to control for population stratification.

### SNPs and Significance Threshold in the Discovery Analysis

Detecting differences in group variances demands larger sample sizes than when detecting differences in group means. Given that our cohort is smaller than the typical sample size of previous vQTL studies, we increased the minimum minor allele frequency (MAF) cutoff for the sake of maintaining statistical power. SNPs with MAF <0.2 were excluded, giving a total of 3,104,639 qualified autosomal SNPs (genotyped and imputed). Because SNPs with MAF >0.2 represent much less of the genome-wide variation than typically interrogated in a GWAS, to reduce burden of multiple comparisons, we used a relaxed threshold of 5 × 10^–7^ to identify suggestive hits for the sake of not missing potential signals. We calculated the empirical type I error rate at the 5 × 10^–7^ level after randomly shuffling the phenotypic data (see section “Results”), and as we expect some false associations, to prioritize SNPs for the G × G interaction stage, we further considered the biological relevance of the loci where the vQTLs are located rather than relying on their *p*-value alone.

### Statistical Methods

We used the Levene’s test with median implemented in the OSCA software package^1^ to identify SNPs associated with the variance of 20 quantitative facial measurements. The Levene’s test is equivalent to a test of differences in mean deviation across subgroups, with the deviation calculated as the absolute difference between an individual’s phenotypic value and their group-specific mean/median. We used the median as the central measure because it provides more robustness to non-normality and outliers than does the mean. To control for covariates, age, age^2^, sex, height, weight, facial size, and four genetic ancestry PCs were first regressed on facial traits in a linear regression model, and the residuals were then used as the phenotype in statistical tests.

The G × G interaction test was done in PLINK (Purcell et al., 2007) with the command –epistasis, which uses a linear regression to model phenotypic residuals on the main effects of each inspected SNP and an interaction term for the pair. Since interaction tests at the vQTL SNP would inevitably lead to a violation of the assumption of variance homogeneity in the generalized linear model, we examined Q–Q plots of the interaction *p*-values, and when there was evidence of inflation (quantified by the genomic inflation factor, λ), we used the robust standard error to obtain unbiased standard errors of the coefficients under variance heteroscedasticity (R package sandwich). This technique was able to correct the inflation and generate well-behaved Q–Q plots. In the discovery analysis, all G × G interaction analyses were performed between a single SNP and all other SNPs across the genome, and therefore a *p*-value below the conventional threshold of 5 × 10^–8^ was considered significant. Co-localization plots for the vQTL analysis and the G × G interaction summary statistics were generated by R package LocusCompareR (Liu et al., 2019).

Although the current study had a primary focus on G × G interactions as the underlying genetic process for variance heterogeneity, we also considered possible gene–environment (G × E) interactions by using a linear regression model in PLINK. Both the 1 degree-of-freedom (*df*) test for the interaction effect only and the 2 *df* test for the main and the interaction effects jointly were used. Only a limited set of environmental factors were collected in our cohort, and we chose to examine the interactions of SNPs with age and sex.

### Replication

The replication cohort consisted of 5,128 unrelated individuals of Asian ancestry collected for the Korean Genome and Epidemiology Study (KoGES) in South Korea from 2009 to 2012 (Ansan–Ansung cohort), for which standardized two-dimensional (2D) frontal photographs were available. The cohort, phenotyping approach, and genotype data have been described in detail previously (Cha et al., 2018). The maximum upper width of the face was represented by the zygion-to-zygion distance (zyR–zyL), which, in a 2D representation, is approximately equivalent to the 3D cranial base width measurement in the discovery sample. ZyR–zyL was pre-adjusted for age, sex, height, and weight and was analyzed using the same statistical approaches applied in the discovery stage. We considered first a SNP-level replication where the exact same SNPs identified in the discovery analysis or, when unavailable, their proxies identified based on LD were tested in the replication data. Next, we sought for a locus-level replication where the SNPs located within 500 kb on either side of the discovery signals were considered. The significance threshold was determined by the Bonferroni method correcting for the effective number of tests (*M*_*eff*_), which was computed according to the eigenvalue-based procedure by Li and Ji (2005), informed by the 1,000 Genomes Project East Asian LD structure. For the locus-level replication of the G × G interaction signal, a *p*-value below 0.05 divided by the product of the *M*_*eff*_ of the two regions under test was considered significant.

### *Prickle1*^*Bj*^ Mouse Husbandry and Sample Preparation

The *Prickle1*^*Bj*^ mouse line is maintained as heterozygotes outbred to wild-type *C57/Bl^6*J*^* individuals. All animal work is approved by the University of Pittsburgh Institutional Animal Care and Use Committee. To collect embryos, we performed timed mating, where the presence of the plug was designated as embryonic day 0.5 (E0.5). On the appropriate day of pregnancy, we euthanized the pregnant dam and collected the embryos *via* caesarian section. We confirmed the embryonic development stage based on morphology. Genotyping was performed using custom SNP Taqman assays (AH7041R, Invitrogen, Waltham, MA, United States).

Embryos were fixed overnight in 4% paraformaldehyde, followed by dehydration and storage in 70% ethanol until further processing. For the tissue sections, the embryos were embedded in paraffin wax, sectioned at 8-μm thickness, and placed on (3-aminopropyl) triethoxysiloxane (TESPA)-treated slides that were stored at 4°C until use.

### Mouse Morphometrics

The superior view images of 7 *Prickle1*^+^*^/^*^+^, *10 Prickle1^*Bj/*^*^+^, and *13 Prickle1^*Bj/Bj*^* heads at E14.5 were captured on a Leica M165FC dissecting microscope with a DFC 450 camera at 1.6× magnification (Leica, Wetzlar, Germany). Care was taken to avoid excess canting of the heads during imaging. Six landmarks were placed around the cranium using 3D Slicer (Fedorov et al., 2012): two bilaterally at the eyes, two bilaterally at the ears, and one each at the midline on the anterior and posterior borders of the cranium. Using MorphoJ (Klingenberg, 2011), the landmark configurations were subjected to generalized Procrustes superimposition in order to translate, rotate, and scale them to unit centroid size and to facilitate relative comparison of cranial base width by genotype. A linear distance capturing cranial base width was calculated between the left and right landmarks placed at the junction of the posterior end of the eye globes where they meet the cranium (Figure 2A); this distance approximated our measure of cranial base width in humans. Linear regression and Levene’s test were then performed to test for differences in the mean and variance, respectively, of cranial base width across the three genotype groups under the additive genetic model while adjusting for litter and centroid size (as a global measure of head size) as covariates. Analysis was performed in the R statistical environment. Differences were considered statistically significant at *p* < 0.05.

### Mouse Immunofluorescence Staining and Imaging

For double immunofluorescence labeling, we used the anti-Focadhesin (HPA055015, Sigma, St. Louis, MO, United States) and the anti-Prickle1 (sc-393034, Santa Cruz, Santa Cruz, CA, United States) antibodies and performed citrate buffer antigen retrieval. After antigen retrieval, the slides were briefly blocked and then incubated overnight in the fridge with the primary antibodies (diluted 1:100). The next day, the slides were washed followed by incubation with secondary antibodies (Alexa Flour, 1:200). The sections were mounted with Prolong Gold with DAPI (Invitrogen). Fluorescent images were captured on a Nikon TE2000 inverted fluorescent microscope (Nikon, Tokyo, Japan) and are represented as a single slice of an image stack. *Prickle1*^+^*^/^*^+^ and *Prickle1*^*Bj/Bj*^ images were captured at the same exposure and magnification.

### Ethics Statement

Institutional ethics (IRB) approval was obtained at each recruitment site (University of Pittsburgh Institutional Review Board #PRO09060553 and #RB0405013; UT Health Committee for the Protection of Human Subjects #HSC-DB-09-0508; Seattle Children’s Institutional Review Board #12107; University of Iowa Human Subjects Office/Institutional Review Board #200912764 and #200710721; and the Korea Institute of Oriental Medicine #I-2007/006-002). All adult subjects gave written informed consent prior to participation; for children, written consent was obtained from a parent or legal guardian. All procedures performed in this study were conducted in accordance with the guidelines of the Declaration of Helsinki. All experimental protocols using mice were approved by the University of Pittsburgh Institutional Animal Care and Use Committee (#17050839) and carried out in accordance with institutional animal care protocols.

## Results

### vQTL Search for 20 Facial Measurements Identified *PRICKLE1*

**FIGURE 1:**
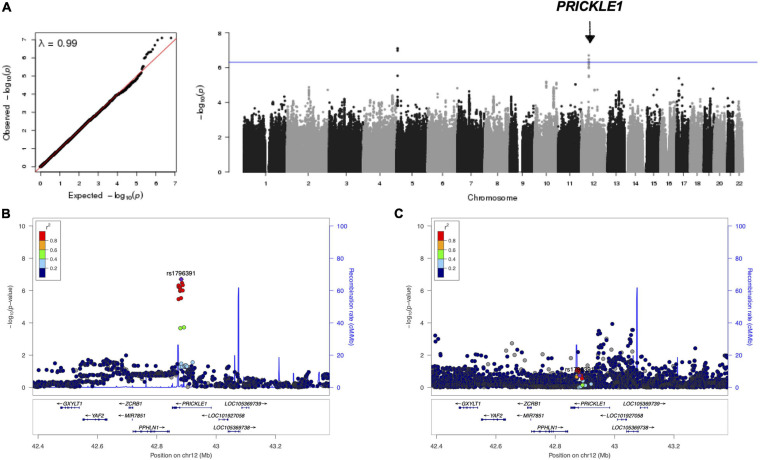
Results of the genome-wide variance quantitative trait locus (vQTL) analysis for cranial base width. **(A)** Q–Q plot and Manhattan plot. There was no sign of inflation. The *arrow* points to the *PRICKLE1* locus on chr12. The *blue horizontal line* indicates suggestive threshold 5 × 10^– 7^. The *bottom two plots* show the association –log_10_(*p*-value) at the *PRICKLE1* locus with the variance **(B)** and the mean **(C)** of cranial base width.

In a cohort of 2,447 unrelated individuals with 3D facial surface data, we conducted a genome-wide search of vQTLs for 20 facial measurements (Supplementary Figure 1 and Supplementary Table 1) using the Levene’s test of medians. No genome-wide significant vQTLs were identified. Ten suggestive loci with *p*-values below 5 × 10^–7^ were observed (Table 1). None of these loci have been implicated in prior facial GWAS or candidate gene association studies. Most of the lead SNPs are intergenic, and the nearby genes are not known to have roles in craniofacial morphogenesis. However, 12q12 showed evidence of association with variance in cranial base width, and its lead SNP rs1796391 was located in an intron of *PRICKLE1* (Figure 1). The Levene’s test statistics for cranial base width were well-behaved and there was no sign of genomic inflation or deflation (Figure 1A). *PRICKLE1*, when disrupted, has been linked to craniofacial malformations, such as orofacial clefting (OFC), in animal models and in humans (Yang et al., 2013, 2014; Wan et al., 2018; Ahsan et al., 2019). Interestingly, excessive cranial base/upper facial width has been reported as a feature in families with a history of OFC (Fraser and Pashayan, 1970; Nakasima and Ichinose, 1983; Blanco et al., 1992; Mossey et al., 1998; Suzuki et al., 1999; Chatzistavrou et al., 2004; McIntyre and Mossey, 2004; Yoon et al., 2004; Weinberg et al., 2006, 2008; Roosenboom et al., 2017).

**TABLE 1:** Suggestive variance quantitative trait loci (vQTLs) of facial features (*p* < 5 × 10^–7^).

Cytoband	Lead SNP	MAF	POS (bp)	*p*-value	Facial distance	Nearest gene
10q25.1	rs11192543	0.36	107,418,528	5.8E−08	OutCanthWidth	*YWHAZP5*
5p15.31	rs495831	0.23	6,551,690	8.0E−08	CranBaseWidth	*LINC01018*
15q12	rs7182802	0.20	27,347,206	1.1E−07	NasalWidth	*GABRG3*
21q22.13	rs73204235	0.25	37,819,218	1.2E−07	LowLipHeight	*CLDN14*
17q21.2	rs34272903	0.35	39,721,357	1.2E−07	NasalHeight	*KRT9*
3q27.2	rs2140287	0.46	185,217,189	1.4E−07	MorphFaceHeight	*TMEM41A*
12q12	rs1796391	0.25	42,882,153	2.0E−07	CranBaseWidth	*PRICKLE1*
16p13.3	rs1492382	0.24	7,030,309	2.6E−07	NasalPro	*RBFOX1*
10q26.2	rs12256165	0.20	130,048,037	3.2E−07	NasalAlaLength	*LINC01163*
4p15.32	rs1522074	0.38	17,170,581	4.6E−07	UpFaceDepth	*MTND5P4*

In light of the potential function of *PRICKLE1* in facial development, we decided to directly test its role using a hypomorphic *Prickle1* mouse allele. The *Prickle1*^*Bj*^ mouse allele has a missense mutation in Prickle1 at c:G482T (p:C161F) (Gibbs et al., 2016; Wan et al., 2018). This mutation causes the substitution of cysteine with a phenylalanine in a cysteine knot that is required for protein–protein interactions. The *Prickle1*^*Bj*^ allele is a hypomorphic allele and bypasses the pre-gastrulation lethality of the conventional knockout allele (Tao et al., 2009). *Prickle1*^*Bj/Bj*^ survivors die shortly after birth due to cardiovascular and craniofacial anomalies (Gibbs et al., 2016; Wan et al., 2018).

**FIGURE 2:**
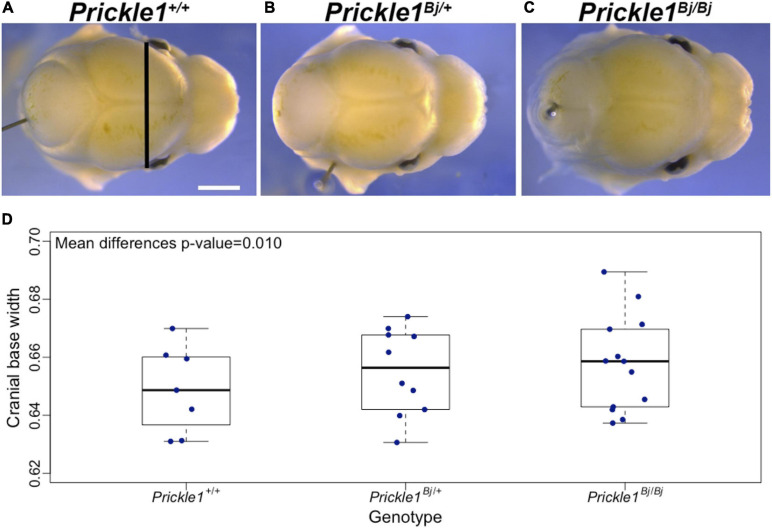
The *Prickle1*^*Bj*^ mouse allele dosage was associated with a wider cranial base. **(A–C)** Superior views of littermates at embryonic day 14.5 showing craniofacial shape changes in *Prickle1*^*Bj/Bj*^ compared to *Prickle1*^+^*^/^*^+^. Cranial base width measurement is indicated by the *black line* in **(A)**. *Scale bar*, 1 mm (and applies to **A–C**). **(D)** Box plot of the cranial base width. *Thick horizontal lines* represent group medians. The *p*-value was obtained from the test of group mean differences assuming an additive genetic model, adjusting for head size and litter.

We explored to what extent the *Prickle1*^*Bj*^ allele affected the cranial base width in a dosage-dependent fashion using data from 30 mice. At E14.5, *Prickle1*^*Bj/Bj*^ mice exhibited significantly wider cranial bases (*p* = 0.01) compared to *Prickle1*^+^*^/^*^+^ and *Prickle1*^*Bj/*+^ littermates (Figure 2). The variance of this phenotype in the three genotype groups was not significantly different (*p* = 0.72), which could be due to a lack of power given the limited number of mice.

We observed 11 SNPs with a *p* < 5 × 10^–7^ after randomly shuffling the cranial base width among our samples (Supplementary Figure 2), indicating that some of the 10 identified suggestive vQTLs are likely spurious associations. We therefore selected *PRICKLE1* rs1796391, the only signal which has been previously studied in craniofacial disorder, as the prioritized locus to be further examined in the G × G interaction stage and did not pursue an analysis of the other nine.

### G × G Interaction of *PRICKLE1* and Cranial Base Width

**FIGURE 3:**
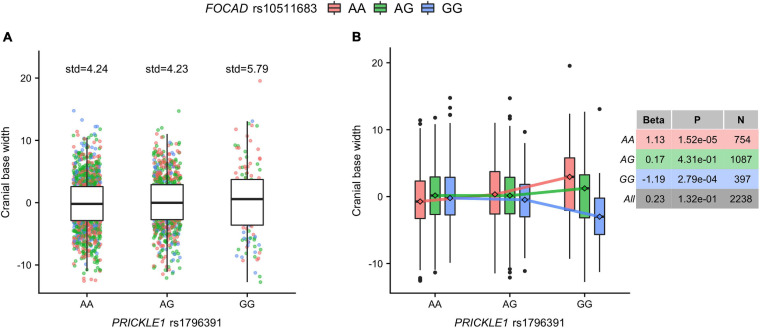
The cranial base width variance heterogeneity at rs1796391 (*PRICKLE1*) was induced by its gene-by-gene (G × G) interaction effect with rs10511683 (*FOCAD*) on phenotypic mean. **(A)** Box plots show the residual of cranial base width [after regressing out age, age^2^, sex, height, weight, facial size, and four genetic principal components (PCs)] in the three genotype groups of the variance quantitative trait locus (vQTL) rs1796391 (*PRICKLE1*), colored by the genotype at the interacting SNP rs10511683 (*FOCAD*). Standard deviations are shown *above boxes*. **(B)** Box plots show the distribution of the phenotypic residual in all nine combinations of the genotypes at rs1796391 (*PRICKLE1*) and rs10511683 (*FOCAD*). The *x*-axis, *y*-axis, and the color scheme are the same as those in **(A)**. Medians are represented by *diamonds* and connected to form segments, the slopes of which indicate the group-specific effects of rs10511683 (*FOCAD*). The non-parallel pattern is the hallmark of statistical interaction. *Table to the right* shows the association (with the mean of cranial base width) beta coefficients, *p*-values, and sample sizes for rs10511683 (*FOCAD*) in subgroups defined by the genotype at rs1796391 (*PRICKLE1*) and the combined sample.

We followed up on the human *PRICKLE1* vQTL to explore the source of the phenotypic variance heterogeneity, with a particular focus on its G × G interaction effect. We utilized the robust standard error (see section “Materials and Methods”) to ensure well-behaved statistics for the genome-wide search of SNPs interacting with the lead *PRICKLE1* SNP rs1796391 (Supplementary Figure 3A). While no SNP pairs reached the genome-wide significance threshold (Supplementary Table 2 and Supplementary Figure 3B), the top interaction signal was seen at the gene *FOCAD* on chromosome 9, with its intronic SNP rs10511683 interacting with the *PRICKLE1* vQTL rs1796391 (*p* = 5.82 × 10^–7^) (Supplementary Figure 3C). Figure 3 illustrates the form of this G × G interaction effect and its relationship with the vQTL signal at the *PRICKLE1* locus. The minor allele homozygote group (Figure 3A, rightmost) of *PRICKLE1* rs1796391 displayed the largest phenotypic variance, which is a result of the differential phenotypic means when stratified by the genotype at the interacting *FOCAD* SNP rs10511683 (Figure 3B). The right table in Figure 3B displays the association effect sizes and the *p*-values of rs10511683 (*FOCAD*) for cranial base width residuals in the unstratified and the stratified data. The minor allele of rs10511683 (*FOCAD*) was associated with a wider cranial base in people with the AA genotype at rs1796391 (*PRICKLE1*), whereas it showed an opposite effect of similar magnitude in the *PRICKLE1* GG group. *FOCAD* SNP rs10511683 had small *p*-values for its association with the phenotypic mean in the stratified analyses of the AA and GG groups of rs1796391 (*PRICKLE1*), but not in the combined sample, where the opposite effects offset each other (*p* = 0.13) (also see Supplementary Figure 3D).

We queried the human cell line experimental data from the Roadmap Epigenomics Project and the ENCODE Project for potential regulatory roles of the identified non-coding SNPs (Supplementary Figure 4). The genomic sequence enclosing rs10511683 (*FOCAD*) overlaps with weak signals of histone modifications, signifying enhancer activity in chondrocyte cells and osteoblast primary cells. SNP rs1796391 (*PRICKLE1*) also showed a potential enhancer signature in osteoblasts. These cell types are important players during craniofacial morphogenesis, and studying cell type-specific roles of *PRICKLE1* and *FOCAD* in future work could potentially provide more insights.

### The Relationship Between Variance Heterogeneity and G × G Interaction at the *PRICKLE1* Locus

**FIGURE 4:**
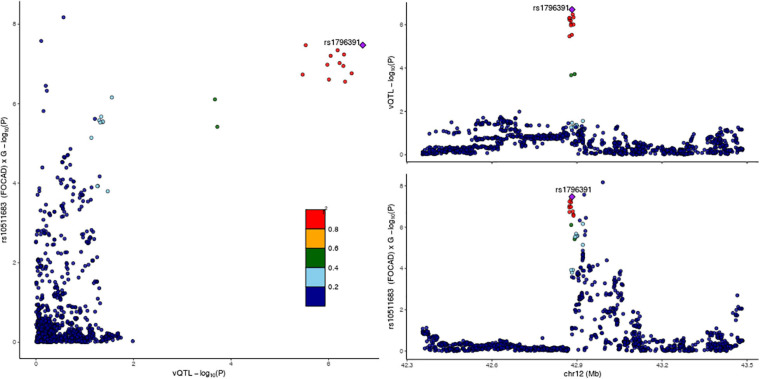
LocusCompare plot at the *PRICKLE1* locus for cranial base width. **(Left)** Joint distribution of the –log_10_(*p*-value) from the Levene’s test and the gene-by-gene (G × G) interaction test, colored by linkage disequilibrium (LD) with rs1796391. Co-localization of the signals is supported by the clustering of *dots at top right*. **(Top right)** –log10(*p*-value) from the Levene’s test on the *y*-axis and genomic coordinate on the *x*-axis. **(Bottom right)** -log_10_(*p*-value) from the G × G interaction test between rs10511683 (*FOCAD*) and SNPs at the *PRICKLE1* locus on the *y*-axis.

We provided two pieces of evidence supporting that this G × G interaction effect can at least partially account for the observed variance heterogeneity. Firstly, a scatter plot with the local LD pattern taken into account demonstrated a co-localization of the vQTL and G × G interaction test summary statistics at the *FOCAD* locus (Figure 4), in agreement with the hypothesis that the interaction effect underlies the observed variance heterogeneity. Secondly, a stratified vQTL analysis was performed for *PRICKLE1* SNPs in each of the three genotype groups defined by rs10511683 (*FOCAD*) separately. If the variance heterogeneity at rs1796391 (*PRICKLE1*) was induced by its interaction with rs10511683 (*FOCAD*), we would expect to see weakened signals in each group. In accordance with this expectation, the group-specific vQTL signals were either absent or much attenuated compared to the unstratified analysis (Supplementary Figure 5). Specifically, the *p*-values of rs1796391 were 1.83 × 10^–4^, 0.05, and 0.6, respectively (Supplementary Table 3). We further downsampled the two larger strata and repeated the analysis in three equally sized groups, the results of which are shown in Supplementary Table 3. We caution that the stratification inevitably led to a reduced power and a tendency for higher variance in smaller groups, which complicated the interpretation of this result. Nonetheless, the results from these two analyses suggest that the interaction with *FOCAD* SNPs is a viable explanation for the observed variance heterogeneity at the *PRICKLE1* locus with no contradicting evidence.

### G × G Interaction of *FOCAD* for Cranial Base Width

**FIGURE 5:**
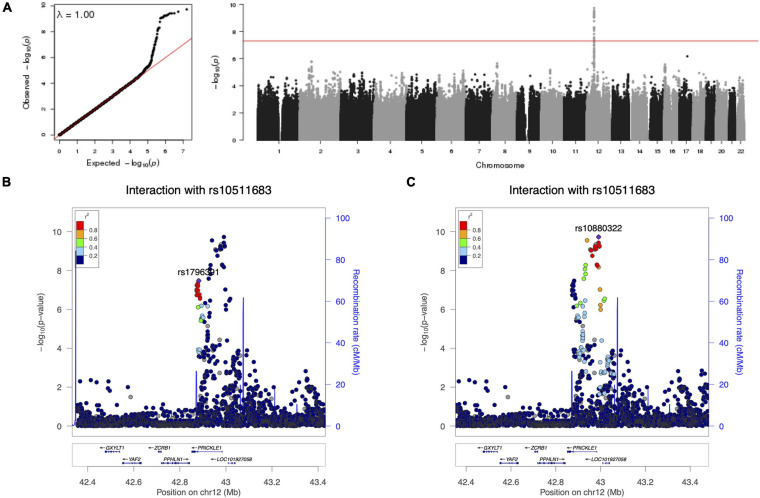
Results of a genome-wide gene-by-gene (G × G) interaction search for loci interacting with *FOCAD* rs10511683. **(A)** Q–Q plot and Manhattan plot. The *red horizontal line* indicates 5 × 10^– 8^. **(B)** Regional association at the *PRICKLE1* locus, colored by the linkage disequilibrium (LD) with the suggestive variance quantitative trait locus (vQTL) rs1796391. **(C)** The same region as in **(B)**, colored by the LD with the other lead SNP rs10880322.

We then turned our focus to *FOCAD* rs10511683 and asked whether it also interacts with other loci. A genome-wide search did not identify significant interacting loci other than *PRICKLE1*. However, we did find a second interaction signal at the *PRICKLE1* locus in close proximity to (7 kb away), but demonstrating low LD (*r*^2^ = 0.11) with the original signal at rs1796391 (Supplementary Table 4 and Figure 5). The lead SNP of this second peak, rs10880322 (*PRICKLE1*), had a significant interaction *p*-value (1.88 × 10^–10^) smaller than that of the original one. SNP rs10880322 had neither a strong mean effect (GWAS *p* = 0.025) nor a variance effect (Levene’s test *p* = 0.428) on cranial base width when tested alone; however, when taking into account its interaction with rs10511683 (*FOCAD*), both the main and interaction effects of rs10880322 became highly significant (Supplementary Table 5 and Supplementary Figure 6). Note that seeing a significant interaction effect with neither SNP being a vQTL, such as the case of rs10880322 × rs10511683, is not a paradox because not all forms of interactions would manifest as variance heterogeneity. We also note that, since the assumption of variance homogeneity was not violated at rs10511683 and there was no sign of inflation in the Q–Q plot (Figure 5A), we need not have used the robust standard error technique in the interaction model, and this gave a smaller *p*-value (3.36 × 10^–8^) for the original rs1796391 (*PRICKLE1*) × rs10511683 (*FOCAD*) interaction than that reported in the section *G × G interaction of PRICKLE1 for cranial base width* (5.82 × 10^–7^) above (where the robust standard error technique was used).

### Other Possible Mechanisms for the Variance Heterogeneity at the *PRICKLE1* Locus

Interaction is only one of the possible explanations for variance heterogeneity; other genetic processes can also give rise to genotype-specific variances. One group of such processes reflects genuine genetic effects, such as the presence of linked causal variants with a mean effect, nearby rare variants with a mean effect, and G × E interactions (Ek et al., 2018). Another group includes statistical artifacts that may confound the analysis and should be managed properly, such as the presence of phenotypic outliers. In our analysis of cranial base width, we noticed a potential outlying measurement for one participant and thus conducted a sensitivity analysis to evaluate the robustness of the signals at rs1796391 (*PRICKLE1*). The outlier is visible in Figure 3A, where one individual in the minor allele homozygous group had the maximum phenotypic residual. After removing this individual, rs1796391 (*PRICKLE1*) yielded a *p* = 9.03 × 10^–7^ in the Levene’s test, and its interaction with rs10511683 (*FOCAD*) had a *p* = 1.07 × 10^–6^ (using robust standard error). Supplementary Figure 7 is a version of Figure 3 with the outlying observation excluded. As expected, both signals were only slightly weakened compared to those in the original analyses, in line with the robustness of the Levene’s test with median to non-normality and outliers.

We further examined alternative explanations involving three different kinds of genetic processes. Firstly, SNP rs1796391 (*PRICKLE1*) did not show a mean effect on cranial base width (*p* = 0.18), nor did any common SNP (MAF > 1%) located within ±500 kb have a small *p*-value (Figure 1C). Secondly, to examine whether there were nearby low-frequency SNPs (0.02% < MAF < 1%) associated with the phenotype, we conducted gene-based tests for *PRICKLE1* coding variants using the sequence kernel association test (SKAT) (Lee et al., 2012) and the combined multivariate and collapsing (CMC) method (Li and Leal, 2008). There was no evidence of association (SKAT *p* = 0.83, CMC *p* = 0.32), although variants that are rarer or undetected could still potentially have an effect. Thirdly, the results from the G × E tests did not support an interaction effect of rs1796391 with either sex or age, which were the only two non-genetic factors available in this cohort (Supplementary Table 6). These results indicated that the genotype-specific variances at the *PRICKLE1* locus were unlikely to be induced by the surrounding mean-controlling SNPs, nor could they be possibly explained by interaction effects with the two non-genetic factors. It should be noted that, theoretically, one cannot exhaust all factors that can lead to heterogeneous variance by assorted mechanisms. For example, just like common and rare SNPs, the genetic mean effect of copy number variants could also confound a variance analysis at nearby loci. Moreover, there are likely plenty of non-genetic factors influencing facial morphology by interacting with individuals’ genetic composition, yet current knowledge is limited and the data necessary for examining how they played a role in our analysis are not available. Therefore, our goal was not to exhaustively scrutinize all possible explanations for the *PRICKLE1* vQTL, but rather to identify the most likely ones as far as the data allow and further verify them in replication and functional experiments.

### Replication Strategy

The replication cohort consisted of 5,128 unrelated individuals of Asian ancestry collected in South Korea for which a suitable measure of maximum upper facial width was available. We conducted both a SNP-level and a locus-level replication of the *PRICKLE1* vQTL signal and the interaction between *PRICKLE1* and *FOCAD*. The SNP-level replication considered the exact same SNPs detected in the discovery stage or, when unavailable, proxy SNPs based on LD. The locus-level replication expanded the genetic markers under consideration using a window size of 500 kb on either side of the genes of interest. We used a minimum MAF of 0.1 for SNP inclusion, which is lower than that of the discovery analysis as the replication cohort is roughly twice as large. For simplicity and clarity, below, we follow a SNP rs identifier with either “*PRICKLE1*” or “*FOCAD*” in parentheses to indicate its locus. Note that this notation does not imply that the SNP falls inside the gene itself, but reflects our locus-level strategy considering a surrounding region (approximately 1 Mb) encompassing the gene. A total of 906 SNPs in and nearby *PRICKLE1* and 796 SNPs in and nearby *FOCAD* were available for replication analysis. The numbers of independent SNPs were estimated to be 138 and 162, respectively, according to the eigenvalue-based procedure by Li and Ji (2005). The significant *p*-value thresholds were therefore set to be 0.05/138 = 3.62 × 10^–4^ for replicating the *PRICKLE1* vQTLs and 0.05/138/162 = 2.24 × 10^–6^ for replicating the *PRCKLE1* × *FOCAD* effect.

### Replication of *PRICKLE1* vQTLs

No *PRICKLE1* locus SNPs yielded a significant replication *p*-value in the Levene’s test (Supplementary Figure 8 and Supplementary Table 7). The discovery lead SNP, rs1796391, was not available in this replication cohort. We identified four proxy SNPs in perfect LD with rs1796391 (*r*^2^ = 1) (Supplementary Table 8), yet none of them showed evidence of influencing phenotypic variance (*p* > 0.1 for all) (Supplementary Table 9).

### SNP-Level Replication of G × G Interactions

Similarly, the lead SNP rs10511683 (*FOCAD*) interacting with *PRICKLE1* in the discovery cohort was not available in the replication cohort. The best proxy SNPs had only moderate LD with rs10511683 (*r*^2^ = 0.63–0.77) (Supplementary Table 8). Supplementary Table 10 displays the G × G interaction test *p*-values between each pair of the proxy SNPs in the two genes. There was no evidence of replication when only these 16 SNP pairs were taken into consideration.

### Locus-Level Replication of G × G Interactions

**FIGURE 6:**
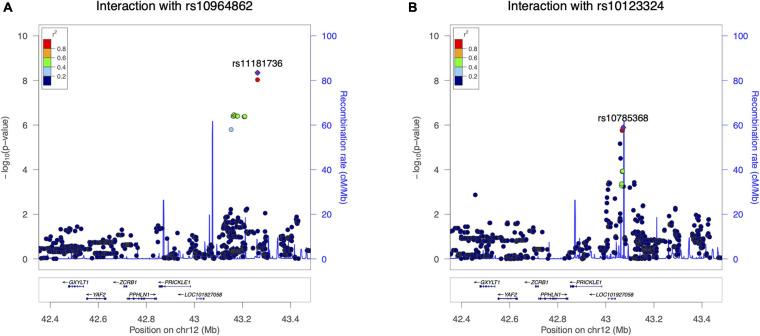
Significant loci in the locus-level replication analysis of *FOCAD* × *PRICKLE1*. **(A)** –log10(*p*-value) of the interaction between rs10964862 (*FOCAD* locus) and single nucleotide polymorphisms (SNPs) at the *PRICKLE1* locus in the replication cohort. **(B)** –log10(*p*-value) of the interaction between *MLLT3* intronic rs10123324 and SNPs at the *PRICKLE1* locus in the replication cohort.

The distribution of the 721K valid *p*-values from the G × G interaction test was consistent with the null expectation, except for those at the tail (Supplementary Figure 9), indicating the presence of significant interactions between the loci at some fine-scale locations. Fifteen pairs of SNPs passed our significance threshold of 2.24 × 10^–6^ (Supplementary Table 11), and these interactions were mainly seen between one of two *FOCAD* locus SNPs (rs10964862 and rs10123324) and the different *PRICKLE1* locus SNPs (Table 2). SNP rs10964862 (*FOCAD*) showed significant interaction with 10 *PRICKLE1* SNPs, and the top two interacting SNP pairs surpassed the conventional genome-wide threshold of 5 × 10^–8^: rs10964862 (*FOCAD*) × rs11181736 (*PRICKLE1*) with *p* = 4.53 × 10^–9^ and rs10964862 (*FOCAD*) × rs11181735 (*PRICKLE1*) with *p* = 9.33 × 10^–9^. These interaction signals were several hundred kilobase pairs away from the original SNP pair identified in the discovery samples (rs1796391 × rs10511683), and the signal at the *PRICKLE1* locus did not overlap with the known coding sequence (Figure 6A). The G × G replication lead SNP rs10964862 (*FOCAD*) is 9.4 kb toward the 5′-UTR of the *IFNW1* gene and overlaps with a sequence showing enhancer signatures in mesenchymal stem cell-derived chondrocyte cells (Supplementary Figure 4). The other SNP at the *FOCAD* locus showing several interactions was rs10123324, an intronic SNP of the *MLLT3* gene. It had the lowest interaction *p*-value with a long non-coding RNA intronic SNP nearby *PRICKLE1*. Signals involving this SNP (rs10123324) were also a few hundred kilobase pairs distant to the target SNP pair (Figure 6B).

**TABLE 2:** Locus-level replication analysis of *FOCAD* × *PRICKLE1* detected significantly interacting SNP pairs between one of the two SNPs (rs10964862 and rs10123324) at the *FOCAD* locus and different *PRICKLE1* SNPs.

*FOCAD* locus					*PRICKLE1* locus Top interacting SNP				Interaction *p*
		
SNP	Gene	Distance from rs10511683 (kb)	MAF	No. of significant interactions	SNP	Gene	Distance from rs1796391 (kb)	MAF	
rs10964862	*IFNW1* 5′-UTR	340	0.138	10	rs11181736	Intergenic	380	0.124	4.53E-09
rs10123324	*MLLT3* intron	345	0.214	5	rs10785368	LINC02451 intron	190	0.430	1.29E-06

*SNP, single nucleotide polymorphism; MAF, minor allele frequency.*

### Replication in the United States Cohort of Interactions With rs10964862 Identified in the South Korean Cohort

We tried to replicate the interacting SNP pairs identified in the South Korean cohort in our United States sample. One of the G × G replication SNPs, rs10123324 (*FOCAD*), failed the quality control procedure in the United States cohort. Since most of the SNPs that the other G × G replication, rs10964862 (*FOCAD*), interacted with (Supplementary Table 11) were unavailable and had no suitable proxy, we conducted a *PRICKLE1* locus-wide test for rs10964862. Out of the 1,405 SNPs tested, the most significant interaction was found for rs10736004 (*PRICKLE1*) with *p* = 6.0 × 10^–4^, which did not pass a Bonferroni threshold correcting for the effective number of tests [120; by the method of Li and Ji (2005)]. Ten SNPs yielding the lowest interaction *p*-values can be found in Supplementary Table 12.

### Co-localization of *Prickle1* and Focadhesin in the Mouse Cranial Base

**FIGURE 7:**
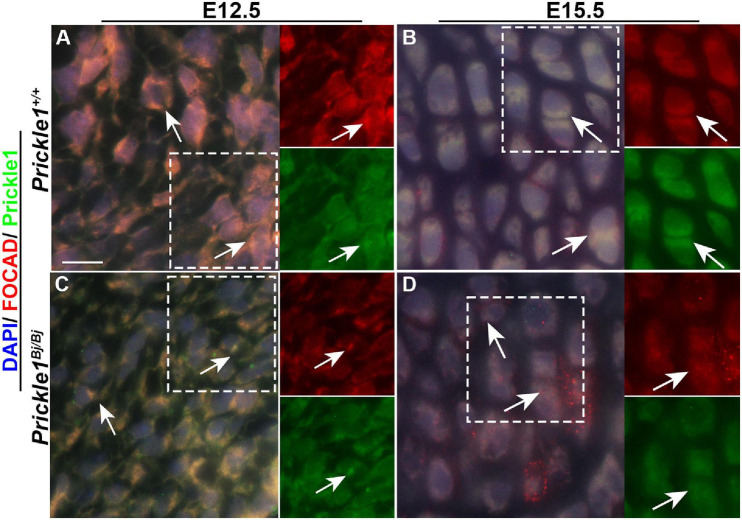
Prickle1 and Focadhesin proteins co-localize in the mouse cranial base mesenchyme. Dual immunofluorescence images of Prickle1 (*green*) and Focadhesin (*red*) proteins, with the nuclei stained with DAPI (*blue*). The *areas within the white boxes* are presented as single channels beside the combined image. **(A,C)** Embryonic day 12.5 cranial bases: in the *Prickle1*^+^*^/^*^+^ mesenchyme, the Prickle1 and Focadhesin proteins are co-localized (*yellow*, *arrows*), while in the *Prickle1*^*Bj/Bj*^ mesenchyme, the signals are co-localized in bright foci, but in the cell periphery. **(B,D)** In *Prickle1*^+^*^/^*^+^ chondrocytes, the signals co-localize (*yellow*, *arrows*) at the cleavage furrow. In *Prickle1*^*Bj/Bj*^ chondrocytes, Focadhesin is found in numerous puncta and in some cleavage furrows.

To locate Prickle1 and Focadhesin (the protein encoded by Focad) in the cranial base, we performed immunofluorescence staining with antibodies to Prickle1 and Focadhesin at E12.5 and E15.5 cranial base. Focad was observed ubiquitously throughout the *Prickle1*^+^*^/^*^+^ cranial base cells at E12.5 (Figure 7A). In contrast, it was enriched in the puncta in the *Prickle1*^*Bj/Bj*^ cranial base (Figure 7C). In the more mature chondrocytes at E15.5, both Prickle1 and Focadhesin proteins were enriched near the cleavage furrow of the *Prickle1*^+^*^/^*^+^ proliferating chondrocytes (Figure 7B). Consistent with the E12.5 results, in the E15.5 *Prickle1*^*Bj/Bj*^ cells, Focadhesin was enriched in the puncta and was not consistently found in the cleavage furrow (Figure 7D). These data suggest that Prickle1 and Focadhesin are co-localized during normal chondrogenesis and that Prickle1 protein contributes to the control of the Focadhesin protein localization during cranial base development.

## Discussion

By focusing on the variance of human facial features, this study uncovered a novel G × G interaction effect between *PRICKLE1* and *FOCAD* in shaping the cranial base/upper facial width and provided strong biological support for this statistical interaction by showing the co-expression of the two genes in relevant craniofacial tissues during early development. Our findings suggest that future genetic studies of human facial morphology should expand their perspective from a sole focus on phenotypic mean to include measures of phenotypic variance and to move beyond the marginal effects of genetic variants in order to investigate their joint and interaction effects.

*PRICKLE1* serves as a signaling factor in the non-canonical Wnt pathway, the disruption of which is known to cause cleft palate and to stunt limb growth (He and Chen, 2012; Mostowska et al., 2012; Yang et al., 2013; Liu et al., 2014; Gibbs et al., 2016; Wan et al., 2018). A recent study characterized the functions of Prickle1a and Prickle1b in zebrafish cranial neural crest cell development during epithelial-to-mesenchymal transition and migration. Studies in mice also support its essential role in craniofacial development. Prickle1 missense allele mutant mice were microcephalic and displayed several craniofacial defects including a cleft lip, incompletely penetrant cleft palate, and a shorter proximal–distal axis of the head (Wan et al., 2018). These phenotypes were a result of the abnormal migration and differentiation of osteoblast precursors in the frontal bone in the absence of a functioning Prickle1 protein. Sequencing studies of patients with craniofacial syndrome implicated both rare and common *PRICKLE1* variants (Yang et al., 2014). In accordance with the facial measurements significantly associated in the current study, an enlarged upper facial width was frequently reported in patients with OFC and their unaffected relatives (Fraser and Pashayan, 1970; Nakasima and Ichinose, 1983; Blanco et al., 1992; Mossey et al., 1998; Suzuki et al., 1999; Chatzistavrou et al., 2004; McIntyre and Mossey, 2004; Yoon et al., 2004; Weinberg et al., 2006, 2008; Roosenboom et al., 2017). Our human data results suggested that *PRICKLE1* may not operate by directly altering the width of the face, but instead *via* its control over how variable the phenotype can be. In the mouse cranial base, we observed the opposite, i.e., the *Prickle1*^*Bj*^ allele had a mean effect, but not a variance effect. This discrepancy may be explained by a combination of developmental timing, inherent species differences, and low power in detecting the variance effect in a small number of mice.

*FOCAD* encodes a focal adhesion complex protein (Focadhesin) (Brockschmidt et al., 2012). There is, so far, little known about its biological function, except that it is a potential tumor suppressor gene highly expressed in brain tissues (Weren et al., 2015). Intriguingly, two recent GWASes by our group in samples of different ancestries both reported genome-wide significant SNPs associated with facial morphology at 9p21.3, which encompasses *FOCAD*. One of the GWASes was performed in an African population, and the lead SNP at 9p21.3 was ∼357 kb upstream of *FOCAD* (Liu et al., 2021). The association signals were seen for the shape variations of the zygoma, nose bridge, and area the surrounding eyes, and the multidimensional representation of these facial regions likely captured some information about the width of the cranial base. The other GWAS, conducted among Europeans, found a significant association at 9p21.3 with the shape of the eye area (Hoskens et al., 2021). In addition to its association with facial variations in healthy populations, *FOCAD* was also suggested to be potentially involved in craniofacial malformation in a study where a parent-of-origin interaction effect between *FOCAD* and maternal smoking was reported for cleft lip with or without cleft palate (Haaland et al., 2019). Our findings about *FOCAD* and the existing research results together provided multiple lines of evidence converging on it being a potential player in craniofacial-related processes and traits.

Our observational results in humans were echoed, to some extent, in the mouse model. The *Prickle1*^*Bj/Bj*^ mice had significantly wider heads and abnormal localization of the Focadhesin protein. These data, together, strongly support the relevance of Prickle1 and Focad and their potential interaction in the development of the cranial base. Proximal distal growth of the cranial base is accomplished by the expansion of the paired synchondroses. Growth plate development involves a process where chondrocytes in a quiescent resting zone mature into proliferating chondrocytes that line up in columns that are oriented in the proximal distal axis of the cranial base (Kronenberg, 2003). In the cranial base, the proliferating chondrocytes initially divide in the medial–lateral axis and then slide into the proximal–distal columns through a process that requires cell adhesion and Wnt/PCP signaling (Romereim et al., 2014). Our observation of the co-localization of Prickle1 and Focadhesin in the cleavage furrow of normal proliferating chondrocytes supports the hypothesis that Prickle1 and Focadhesin determine the angle of cell division. The *Prickle1*^*Bj*^ allele affects protein function (Gibbs et al., 2016; Wan et al., 2018). Furthermore, our immunofluorescence data suggest that Prickle1 and Focadhesin may interact in this process. Future studies are needed to further elucidate the mechanism by which the two proteins operate together during craniofacial development.

In humans, despite the lack of variance effect of *PRICKLE1* in the replication cohort, we achieved a locus-level replication of its interaction with *FOCAD.* The failure of our SNP-level replication was not surprising. Considering the two cohorts of different ancestries, differences in the LD structure, the non-availability of the discovery lead SNPs, and the differences in the genetic and environmental backgrounds may all have a role in shaping the distribution of the variance and the interaction effects and/or our ability to detect them. These factors may also explain why the interaction signals were detected at different locations relative to *PRICKLE1* and *FOCAD* in the two cohorts—introns of the genes in discovery and outside of the genes in replication. The statistical signals in the two cohorts may implicate the same underlying biology, or they may in fact reflect different mechanisms through which genetic variants influence a facial feature. There was some evidence from existing epigenomic ChIP-Seq data that the non-coding regions with which the lead interacting SNPs overlapped may function as enhancers in relevant cell types (Supplementary Figure 4). Several major links need to be further established, including how the SNPs identified disrupt those enhancers, whether those enhancers target *PRICKLE1* and *FOCAD*, and how *PRICKLE1* and *FOCAD* together affect early craniofacial development. Despite these unknowns, the results from our mouse experiments provided a good reason to hypothesize that, even if different regulatory elements were implicated in the discovery and the replication analysis, these elements likely all targeted *PRICKLE1* and *FOCAD*.

Although the statistical results of our human data that initially led to the experimental interrogation of *Prickle1* and *Focad* were not decisively strong, the fact that we found strong evidence for a genuine biological interaction indicates that the lack of significance was likely due to the limited power. It is well recognized that detecting differences in group variances demands larger sample sizes than when detecting differences in group means. Given that a previous GWAS in the same cohort identified only a limited number of significant loci (Shaffer et al., 2016), we did not expect to see more than a few significant vQTLs. Indeed, the most significant vQTLs in our analysis were only at the suggestive level (5 × 10^–7^). It is a limitation that we followed up on a strictly speaking non-significant signal (*PRICKLE1*), and in the next step of the analysis, its interaction with *FOCAD* again did not reach the genome-wide significance threshold. However, three things together motivated us to pursue a replication and mouse experiments. Firstly, the suggestive *p*-values of a true positive might be a mere result of insufficient power. Secondly, the disruption of *PRICKLE1* was known to associate with craniofacial phenotypes, but nearby SNPs were never significant in the GWAS, implying a role of a rare mutation or a complicated mode of action, such as interaction. Thirdly, the genome-wide search of loci interacting with *FOCAD* did identify *PRICKLE1* as the most and only significant locus (*p* = 1.88 × 10^–10^).

The strategy of variance prioritization showed a clear benefit in studying G × G interactions in the present study. With a sample size of 2,447 individuals, an exhaustive search for pairwise interactions between genome-wide SNPs would not have sufficient power given the huge burden of multiple comparisons, even with the scope restricted to SNPs with a MAF >0.2. As *PRICKLE1* and *FOCAD* were not known to relate in any way before this study, few other pre-selection strategies would have been able to sort them out and make a discovery. Note that the significant rs10880322 (*PRICKLE1*) × rs10511683 (*FOCAD*) interaction exemplified how interaction effects need not cause variance heterogeneity at either locus. Likewise, variance heterogeneity can arise from genetic processes other than interaction, as well as statistical artifacts such as outliers. We carefully scrutinized alternative explanations for the *PRICKLE1* vQTL (see section “Results”), ensuring that, although not impossible, they were not likely to explain our finding. Despite the lack of definite relationship between heterogeneous variance and interaction, a detected vQTL does signify a possible presence of unmodeled statistical interaction, which can be further examined relatively easily. Our results highlighted the potential of vQTL analysis in revealing G × G interactions and underscored the need to explore how genetic factors crosstalk in facial genetic studies.

## Data Availability Statement

Publicly available datasets were generated for this study. These data can be found here: the genetic data and the 20 facial distances evaluated in the United States cohort are available to the research community through the dbGaP controlled-access repository (https://dbgap.ncbi.nlm.nih.gov/) at accession phs000949.v1.p1. The raw source data for the phenotypes – the 3D facial surfaces – are available for the 3D Facial Norms dataset through the FaceBase Consortium (www.facebase.org). The genotypic and phenotypic data of the Korean sample is available upon request from the Korean Genome and Epidemiology Study (KoGES) of the Center for Disease Control and from the Korea Medicine Data Center of the Korea Institute of Oriental Medicine, respectively. The mouse data is available upon request from HSR.

## Ethics Statement

The studies involving human participants were reviewed and approved by University of Pittsburgh Institutional Review Board #PRO09060553 and #RB0405013; UT Health Committee for the Protection of Human Subjects #HSC-DB-09-0508; Seattle Children’s Institutional Review Board #12107; University of Iowa Human Subjects Office/Institutional Review Board #200912764 and #200710721; and the Korea Institute of Oriental Medicine #I-2007/006-002. Written informed consent to participate in this study was provided by the participants’ legal guardian/next of kin. The animal study was reviewed and approved by University of Pittsburgh Institutional Animal Care and Use Committee (#17050839).

## Author Contributions

DL, SW, and JS conceived and designed the study. EF, MM, SW, and JS provided funding for data collection. JH, GW, and LM provided the discovery human data. H-JB and SC provided the replication human data and involved in the replication with Korean subjects. DL spearheaded the statistical analysis and wrote the manuscript. H-JB and ML contributed to the statistical analysis. AE and HS-R conducted the mouse experiments. All authors reviewed and approved the final manuscript.

## Conflict of Interest

The authors declare that the research was conducted in the absence of any commercial or financial relationships that could be construed as a potential conflict of interest.

## Publisher’s Note

All claims expressed in this article are solely those of the authors and do not necessarily represent those of their affiliated organizations, or those of the publisher, the editors and the reviewers. Any product that may be evaluated in this article, or claim that may be made by its manufacturer, is not guaranteed or endorsed by the publisher.

## Footnotes

1
http://cnsgenomics.com/software/osca
