# Supplementary material for: PRICKLE1 × FOCAD Interaction Revealed by Genome-Wide vQTL Analysis of Human Facial Traits
Source: Front Genet. 2021 Aug 9;12:674642. doi: 10.3389/fgene.2021.674642 (PMC8381734; doi:10.3389/fgene.2021.674642)
Supplement: Supplementary file 1 [file Data_Sheet_1.PDF]

Supplemental figures and tables

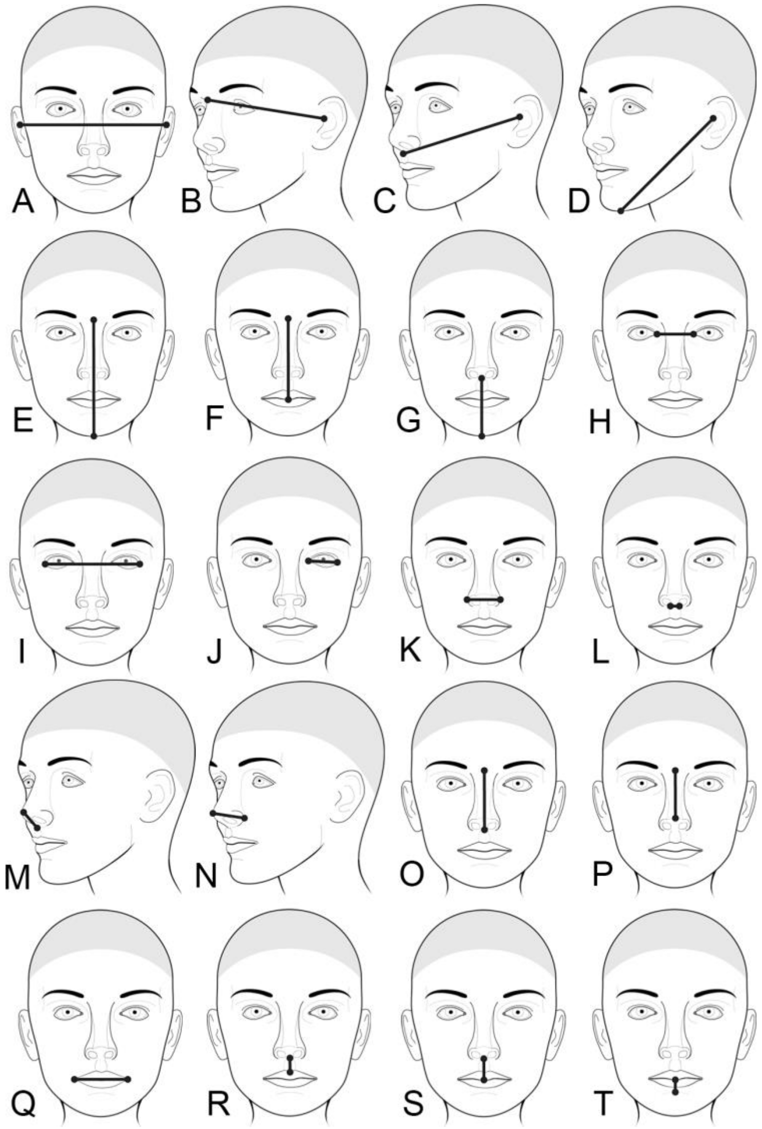

Figure S1: Set of 20 linear distance measurements used in the current study. (A) Cranial base width, (B) Upper facial depth*, (C) Middle facial depth*, (D) Lower facial depth*, (E) Morphological facial height, (F) Upper facial height, (G) Lower facial height, (H) intercanthal width, (I) Outercanthal width, (J) Palpebral fissure length*, (K) Nasal width, (L) Subnasal width, (M) Nasal Protrusion, (N) Nasal ala length*, (O) Nasal height, (P) Nasal Bridge Length, (Q) Labial fissure length, (R) Philtrum length, (S) Upper lip height, and (T) Lower lip height. Measurements with an asterisk (*) are bilateral, but only the left side is shown in the figure. Reproduced from Shaffer et al 2016 [1].

1. Shaffer, J. R., Orlova, E., Lee, M. K., Leslie, E. J., Raffensperger, Z. D., Heike, C. L., et al. (2016). Genome-Wide Association Study Reveals Multiple Loci Influencing Normal Human Facial Morphology. PLoS Genetics, 12(8), e1006149–21.

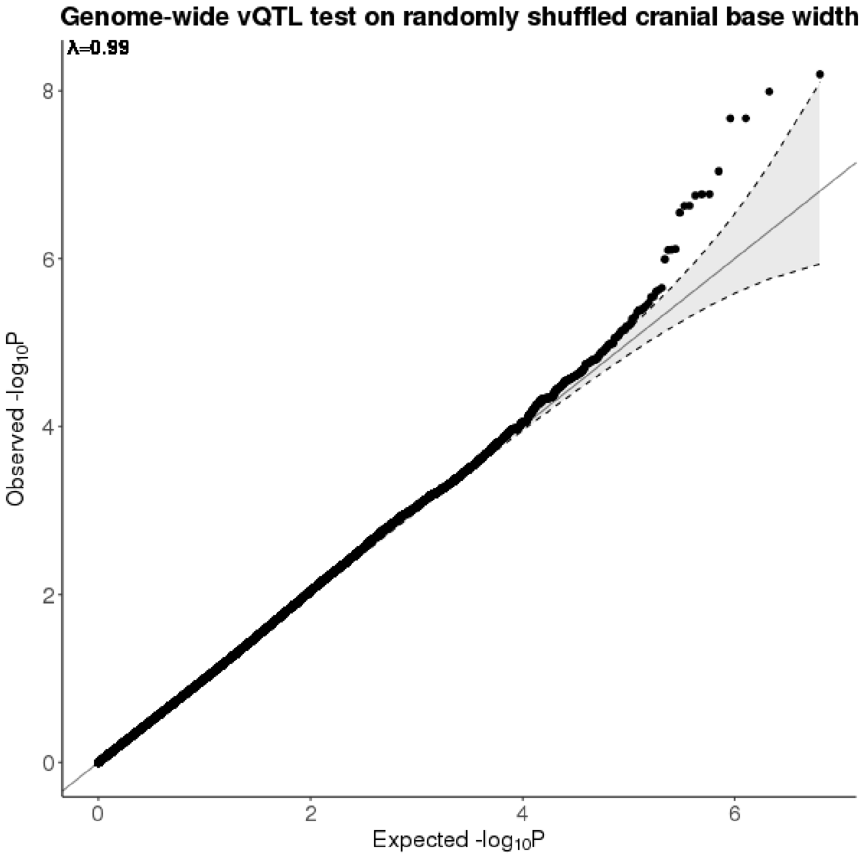

Figure S2: QQ plot of the genome-wide vQTL test p-value after the cranial base width data was randomly shuffled. The genomic inflation factor is indicated in the top left conner. There are 11 p-values $< 5 \times 10^{-7}$.

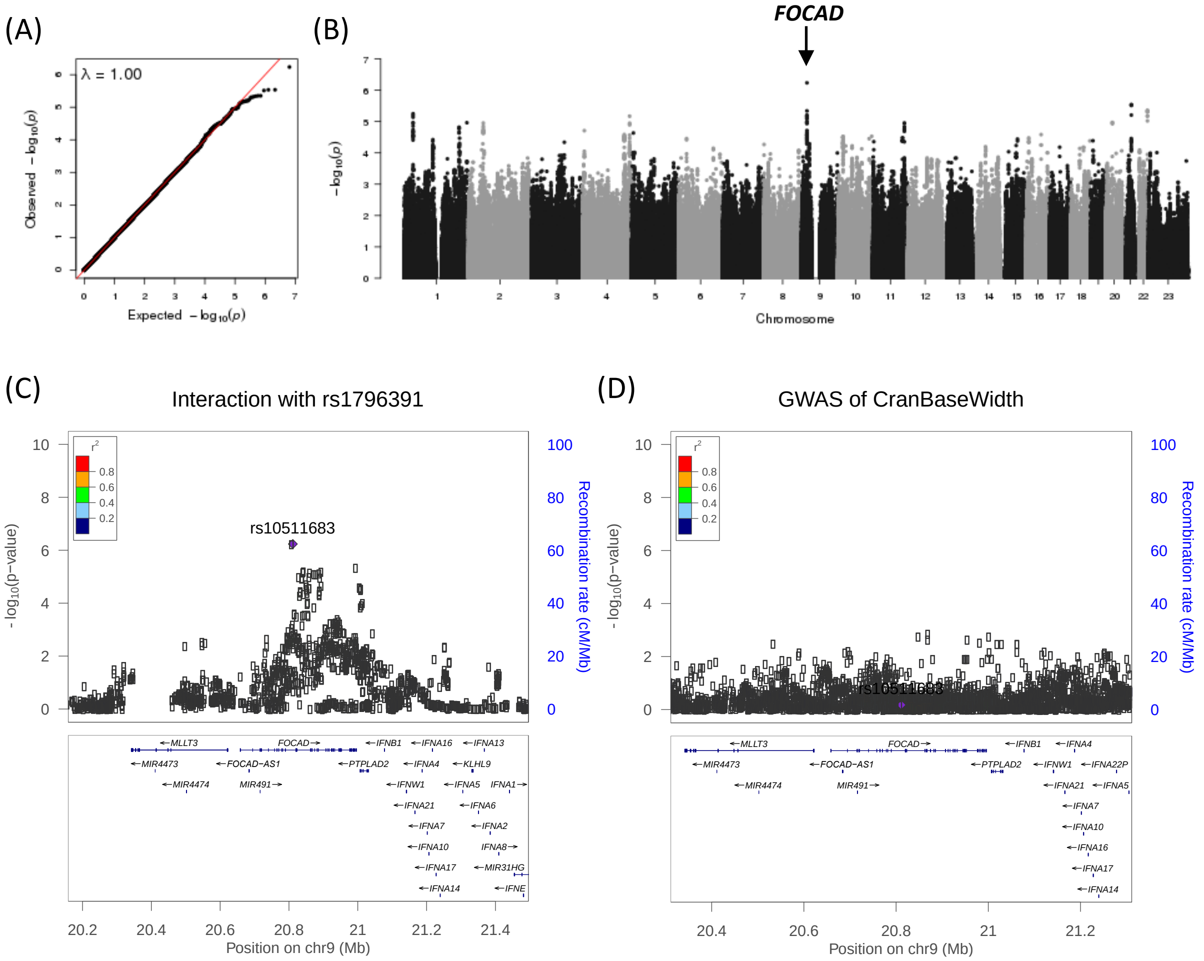

Figure S3: Results of the genome-wide G x G search for loci interacting with *PRICKLE1* rs1796391, using robust standard error. (A) Q-Q plot. (B) -$\log_{10}$(p-value) along genomic locations. The arrow points to the *FOCAD* locus on chromosome 9. (C) Interaction -$\log_{10}$(p-value) at the *FOCAD* locus, colored by the LD with the lead SNP rs10511683. (D) GWAS -$\log_{10}$(p-value) at the *FOCAD* locus, colored by the LD with the lead SNP rs10511683.

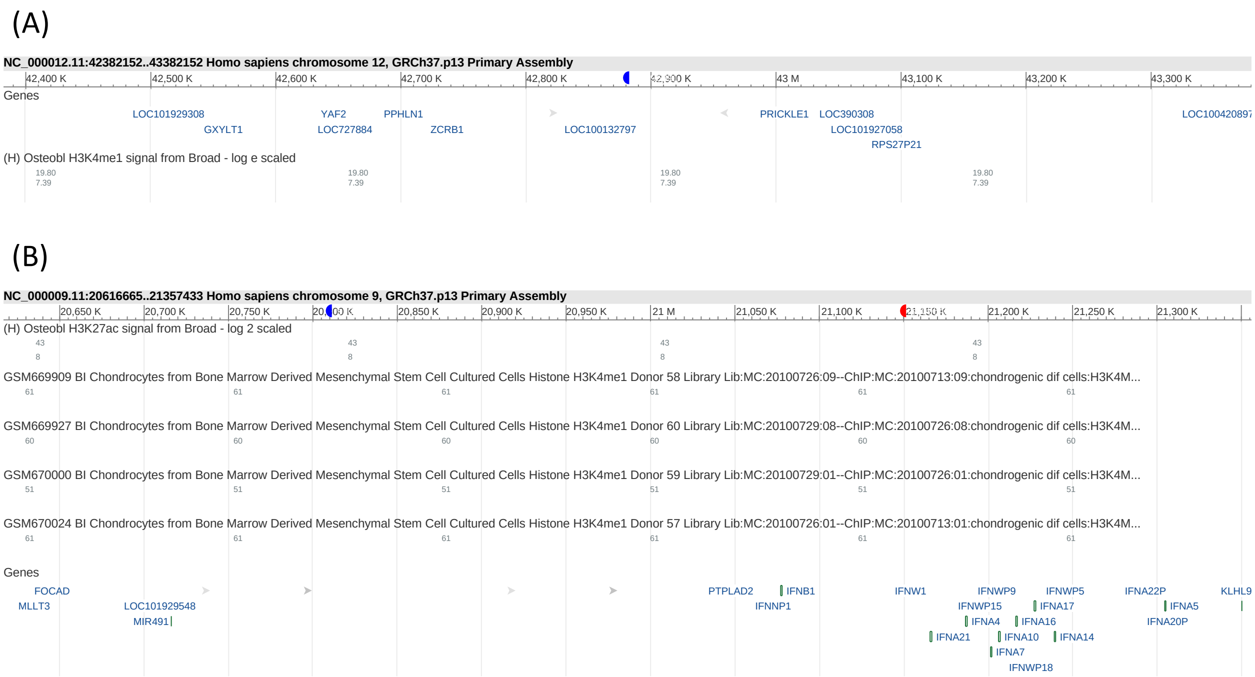

Figure S4: Histone modification ChIP-Seq signals retrieved from the Roadmap Epigenomics Project and the ENCODE project. Vertical red and blue lines denote the position of SNPs of interest. (A) *PRICKLE1* rs1796391 overlaps with H3K4me1 signals in osteoblasts. (B) Blue vertical line: *FOCAD* rs10511683 overlaps with H3K4me1 signals in mesenchymal stem cell derived chondrocyte cells, and H3K27ac signals in osteoblast primary cells. Red line: rs10964862 (downstream *FOCAD*) overlaps with a H3K4me1 peak in mesenchymal stem cell derived chondrocyte cells.

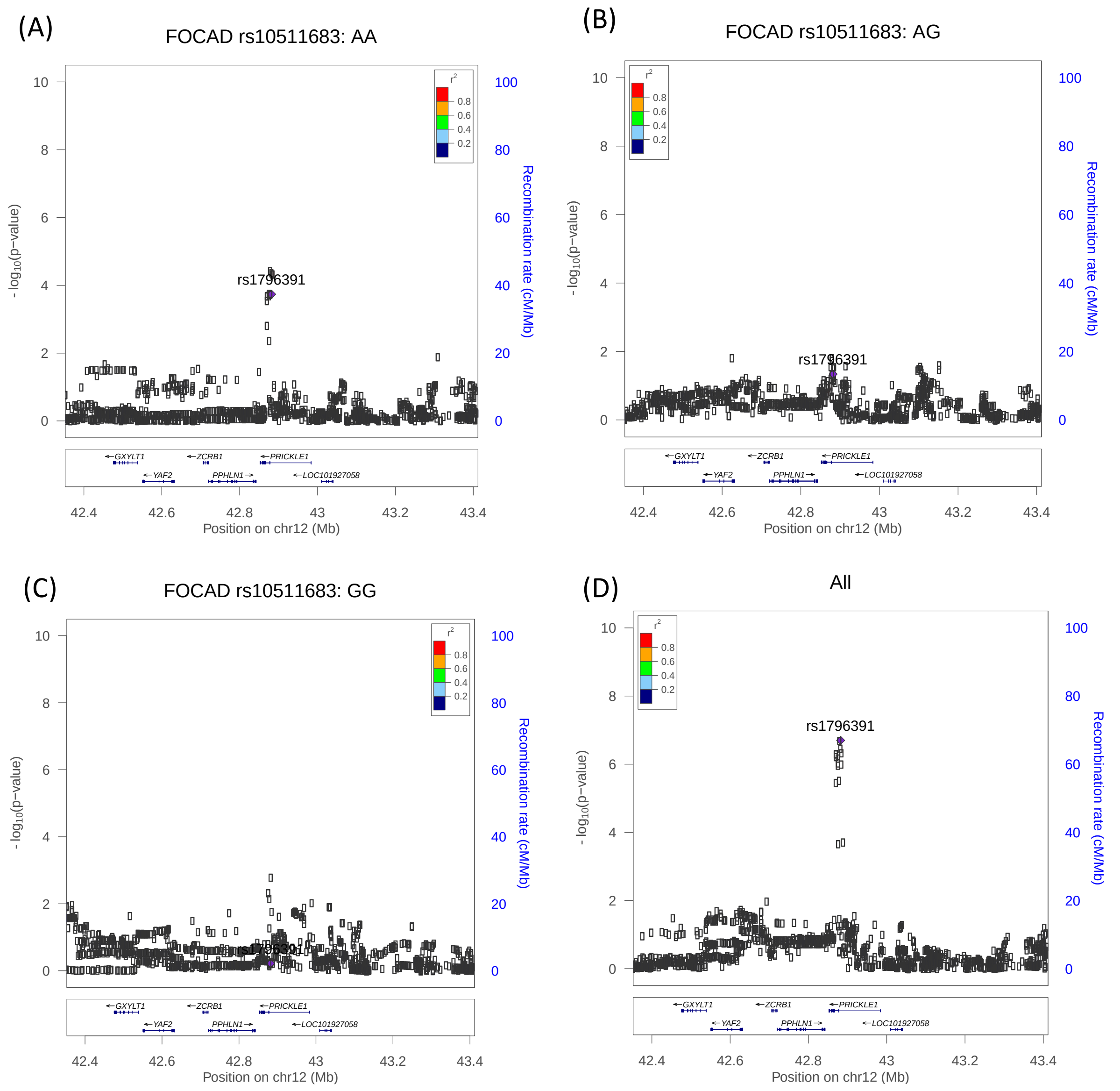

Figure S5: Stratified vQTL analysis of the *PRICKLE1* locus by *FOCAD* rs10511683 genotypes. (A-C) -log10(p-value) in the AA, AG, and GG group. (D) Marginal association -log10(p-value) in the combined sample, same plot as Figure 1C.

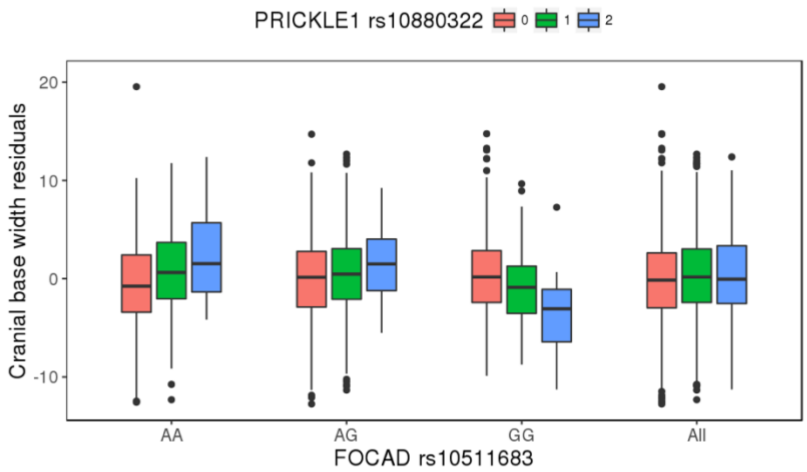

Figure S6: Interaction between *PRICKLE1* rs10880322 and *FOCAD* rs10511683 for cranial base width. SNP rs10880322 had a positive association with cranial base width when the genotype at rs10511683 was AA (leftmost cluster), and a negative association when the genotype at rs10511683 was GG (third cluster). The opposite effects offset in the entire sample (rightmost cluster).

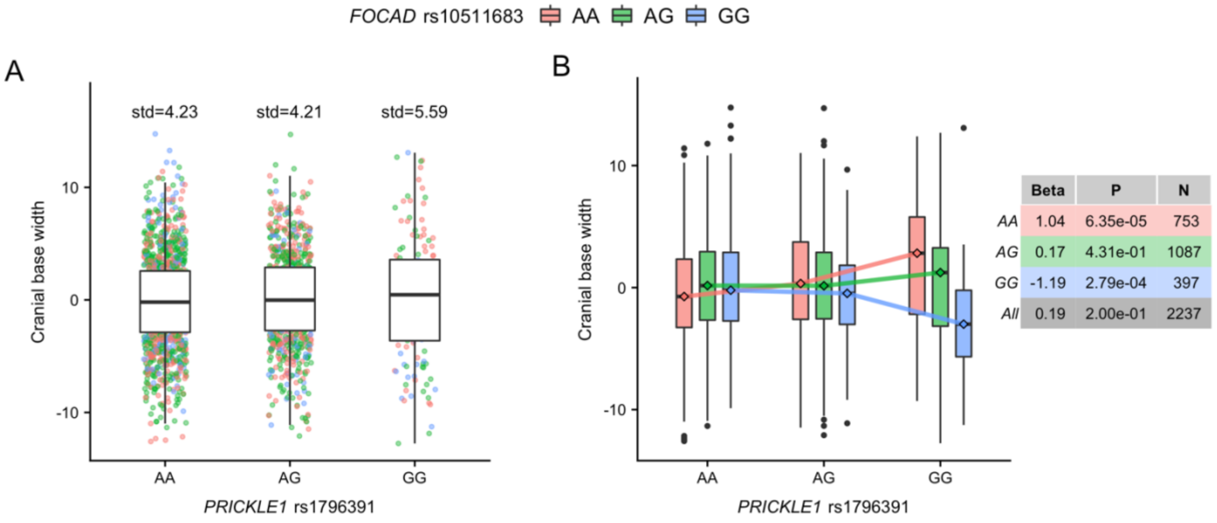

Figure S7: Sensitivity analysis with the outlier individual removed. The signals at rs1796391 (*PRICKLE1*) were robust and did not show a radical change. The layout and interpretation of the figure are the same as in Figure 3. (A) Boxplots show the residual of cranial base width (after regressing out age, age^2, sex, height, weight, facial size, and four genetic PCs) in the three genotype groups of the vQTL rs1796391 (*PRICKLE1*), colored by the genotype at the interacting SNP rs10511683 (*FOCAD*). Standard deviations are shown above boxes. (B) Boxplots show the distribution of the phenotypic residual in all nine combinations of the genotypes at rs1796391 (*PRICKLE1*) and rs10511683 (*FOCAD*). X-axis, Y-axis and the color scheme are the same as in (A). Medians are represented by diamonds and connected to form segments, the slopes of which indicate the group-specific effects of rs10511683 (*FOCAD*). The non-parallel pattern is the hallmark of statistical interaction. Table to the right shows the association (with the mean of cranial base width) beta coefficients, p-values and sample sizes for rs10511683 (*FOCAD*) in subgroups defined by the genotype at rs1796391 (*PRICKLE1*) and the in the combined sample.

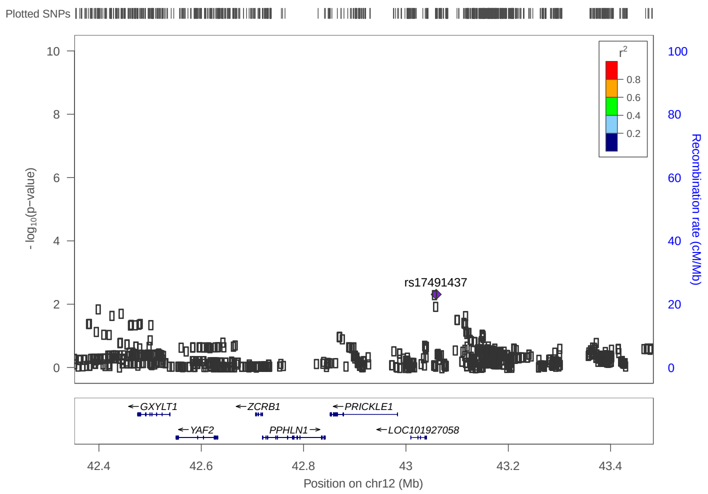

Figure S8: -$\log_{10}$(p-value) of the replication analysis of the *PRICKLE1* vQTL signal

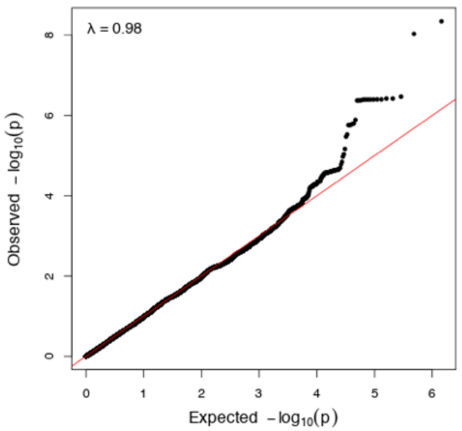

Figure S9: Q-Q plot showing no sign of inflation in the distribution of all 721K pairwise interaction p-values in the replication cohort.

Table S1. List of linear distance measurements

**Measurement**	**Figure** [a]	**Region**	**Landmarks involved**
Cranial base width	Figure S1A	Head	Right Tragion (t_r) - Left Tragion (t_l)
Upper facial depth [b]	Figure S1B	Face	Nasion (n) - Left Tragion (t_l)
Middle facial depth [b]	Figure S1C	Face	Subnasale (sn) - Left Tragion (t_l)
Lower facial depth [b]	Figure S1D	Face	Gnathion (gn) - Left Tragion (t_l)
Morphological facial height	Figure S1E	Face	Nasion (n) - Gnathion (gn)
Upper facial height	Figure S1F	Face	Nasion (n) - Stomion (sto)
Lower facial height	Figure S1G	Face	Subnasale (sn) - Gnathion (gn)
Intercanthal width	Figure S1H	Eye	Right Endocanthion (en_r) - Left Endocanthion (en_l)
Outercanthal width	Figure S1I	Eye	Right Exocanthion (ex_r) - Left Exocanthion (ex_l)
Palpebral fissure length [b]	Figure S1J	Eye	Left Endocanthion (en_l) - Left Exocanthion (ex_l)
Nasal width	Figure S1K	Nose	Right Alare (al_r) - Left Alare (al_l):
Subnasal width	Figure S1L	Nose	Right Subalare (sbal_r) - Left Subalare (sbal_l)
Nasal protrusion	Figure S1M	Nose	Subnasale (sn) - Pronasale (prn)
Nasal ala length [b]	Figure S1N	Nose	Left Alar Curvature Point (ac_l) - Pronasale (prn)
Nasal height	Figure S1O	Nose	Nasion (n) - Subnasale (sn)
Nasal bridge length	Figure S1P	Nose	Nasion (n) - Pronasale (prn)
Labial fissure width	Figure S1Q	Mouth	Right Chelion (ch_r) - Left Chelion (ch_l)
Philtrum length	Figure S1R	Mouth	Subnasale (sn) - Labiale Superius (ls)
Upper lip height	Figure S1S	Mouth	Subnasale (sn) - Stomion (sto)
Lower lip height	Figure S1T	Mouth	Stomion (sto) - Sublabiale (sl)

[a] Measurements shown in Figure S1, parts A-T

[b] indicates bilateral measurements. Further definitions can be found here: https://www.facebase.org/facial_norms/notes/

Table S2. Top ten signals from the genome-wide search of SNPs interacting with *PRICKLE1* rs1796391

SNP1	SNP2 interacting with SNP1			Interaction P
	SNP2	CHR:POS	MAF	
rs1796391	rs10511683	9:20811210	0.421	5.82E-07
12:42882153	rs9977155	21:35231531	0.389	2.91E-06
MAF=0.246	rs1109847	21:35229568	0.431	2.92E-06
PRICKLE1 intron	rs13047166	21:35233984	0.431	3.06E-06
	rs36103341	22:49492120	0.344	4.45E-06
	rs34754534	22:49492130	0.344	4.45E-06
	rs10757169	9:20997340	0.457	4.57E-06
	rs9616427	22:49491796	0.345	4.71E-06
	rs60678220	22:49492066	0.355	4.91E-06
	rs35662237	22:49492241	0.344	4.95E-06

Table S3. Stratified vQTL analysis of *PRICKLE1 rs1796391* by *FOCAD* rs10511683 genotypes, before and after downsampling larger groups to be of the same size as the smallest group

Original					
	FOCAD rs10511683	*PRICKLE1* rs1796391			
		MAF	F-statistic	Levene's P	Group size
	AA	0.256	8.71	1.83E-04	754
	AG	0.238	3.10	0.05	1087
	GG	0.251	0.51	0.60	397
	Unstratified	0.246	16.37	2.00E-07	2303
Down sample					
	FOCAD rs10511683	*PRICKLE1* rs1796391			
		MAF	F-statistic	Levene's P	Downsampled group size
	AA	0.247	4.85	8.32E-03	397
	AG	0.221	0.54	0.58	397
	GG	0.251	0.51	0.60	397

Table S4. Genome-wide significant interactions for *FOCAD* SNP rs10511683

SNP1	SNP2 (all are *PRICKLE1* SNPs)				Interaction P
	SNP2	CHR:POS	MAF	Beta	
rs10511683	rs10880322	12:42991202	0.186	-1.475	1.88E-10
9:20811210	rs11181548	12:42939576	0.169	-1.533	2.83E-10
MAF=0.421	rs12370911	12:42990645	0.187	-1.446	3.75E-10
FOCAD intron	rs11181570	12:42990930	0.187	-1.446	3.82E-10
	rs11181571	12:42990936	0.187	-1.446	3.82E-10
	rs72379663	12:42978342	0.200	-1.403	4.59E-10
	rs10785351	12:42995211	0.184	-1.448	5.51E-10
	rs11181568	12:42975498	0.196	-1.413	5.75E-10
	rs1922758	12:42976258	0.196	-1.413	5.75E-10
	rs11181575	12:42994694	0.183	-1.448	5.83E-10
	rs11181567	12:42974374	0.197	-1.400	7.51E-10
	rs7958546	12:42979737	0.196	-1.399	7.86E-10
	rs140669010	12:42954009	0.195	-1.403	8.29E-10
	rs141455369	12:42973643	0.196	-1.401	8.32E-10
	rs187217275	12:42956s825	0.195	-1.402	8.96E-10
	rs12229911	12:42957775	0.195	-1.400	9.34E-10
	rs11181563	12:42963124	0.197	-1.368	1.74E-09
	rs11837158	12:42983762	0.195	-1.335	5.01E-09
	rs535764464	12:42983324	0.195	-1.332	5.23E-09
	rs7302704	12:42933230	0.192	-1.342	5.26E-09
	rs10880320	12:42985734	0.195	-1.330	5.66E-09
	rs10785350	12:42991138	0.235	-1.225	6.72E-09
	rs11181546	12:42930317	0.194	-1.315	8.50E-09
	rs7316200	12:42932928	0.194	-1.293	1.52E-08
	rs10880312	12:42925175	0.208	-1.248	2.64E-08
	rs1796391	12:42882153	0.246	-1.138	3.36E-08
	rs1669915	12:42881594	0.254	-1.133	3.37E-08
	rs1669917	12:42878100	0.249	-1.120	4.52E-08

Table S5. Marginal, conditional, and interaction effect of *PRICKLE1* rs10880322

Model	Effect	Beta	P
Y ~ rs10880322			
	Marginal	0.37	0.025
Y ~ rs10880322 + rs10511683 + rs10880322 × rs10511683			
	Conditional main	1.64	4.25E-10
	Interaction	-1.48	2.26E-10
	Conditional main + Interaction	-	1.70E-10

Table S6. G × E test p-values for *PRICKLE1* SNP rs1796391

E	Effect under test*	p-value
Sex	GxE	0.80
	G+GxE	0.42
Age	GxE	0.79
	G+GxE	0.42

Table S7. Top ten SNPs from the replication analysis of the *PRICKLE1* vQTL signal

SNP	CHR:POS	MAF	P-value
rs17491437	12:43059128	0.203	0.0049
rs17569596	12:43061380	0.189	0.0115
rs34629101	12:42401839	0.206	0.0138
rs17224156	12:42446725	0.202	0.0190
rs1920685	12:42428674	0.204	0.0222
rs7311360	12:43119740	0.152	0.0226
rs7978127	12:43103039	0.143	0.0296
rs2203735	12:43103728	0.143	0.0301
rs61926670	12:43105423	0.148	0.0347
rs142651303	12:43116544	0.153	0.0368

Table S8. Proxy SNPs for rs1796391 (*PRICKLE1*) and rs10511683 (*FOCAD*) in the replication cohort

Target SNP	Proxy SNP	CHR:POS	MAF	LD with target SNP (r^2)
PRICKLE1 rs1796391	rs1796362	12:42873184	0.221	1
	rs1796361	12:42873402	0.221	1
	rs2708068	12:42873478	0.224	1
	rs1669916	12:42878879	0.209	1
FOCAD rs10511683	rs4978017	9:20739231	0.333	0.631
	rs10757149	9:20831915	0.348	0.767
	rs2026994	9:20834837	0.348	0.647
	rs2151001	9:20836862	0.293	0.697

Table S9. Levene's test results for the proxy SNPs of *PRICKLE1* rs1796391 in the replication cohort

SNP	Beta	P-value
rs1796362	-0.039	0.104
rs1796361	-0.039	0.102
rs2708068	-0.039	0.102
rs1669916	-0.038	0.123

Table S10. G x G test p-values for the proxy SNP pairs in the replication cohort

		PRICKLE1 proxy SNP			
		rs1669916	rs1796361	rs1796362	rs2708068
FOCAD proxy SNP	rs10757149	0.257	0.248	0.247	0.249
	rs2026994	0.268	0.261	0.260	0.260
	rs2151001	0.146	0.151	0.150	0.166
	rs4978017	0.264	0.242	0.239	0.227

Table S11. Significant G × G in the replication cohort

FOCAD			*PRICKLE1*			Interaction P
SNP	CHR:POS	MAF	SNP	CHR:POS	MAF	
rs10964862	9:21151553	0.138	rs11181736	12:43262513	0.124	4.53E-09
			rs11181735	12:43262480	0.139	9.33E-09
			rs76090145	12:43165077	0.102	3.42E-07
			rs74615597	12:43168457	0.102	3.81E-07
			rs80288071	12:43169982	0.102	3.81E-07
			rs12580440	12:43179198	0.102	4.00E-07
			rs76815337	12:43180097	0.102	4.00E-07
			rs76387369	12:43176684	0.102	4.02E-07
			rs17091875	12:43160470	0.102	4.03E-07
			rs17091890	12:43161655	0.102	4.03E-07
rs10123324	9:20466105	0.214	rs10785368	12:43072515	0.430	1.29E-06
			rs12372464	12:43069295	0.427	1.63E-06
			rs7309687	12:43068644	0.429	1.71E-06
			rs11181632	12:43069110	0.427	1.74E-06
			rs4288814	12:43067451	0.430	1.75E-06

Table S12. Top ten interaction signals involving rs10123324 in the discovery cohort

SNP1	SNPs at the *PRICKLE1* locus				Interaction P
	SNP2	CHR:POS	MAF	Beta	
rs10964862 MAF=0.330	rs10736004	12:43042979	0.443	-0.682	6.28E-04
	rs7305335	12:43042729	0.442	-0.677	7.01E-04
	rs7305361	12:43042784	0.442	-0.677	7.02E-04
	rs11403754	12:43042271	0.443	-0.668	8.34E-04
	rs7135771	12:43042869	0.441	-0.665	8.52E-04
	rs10748335	12:43042065	0.442	-0.663	9.08E-04
	rs2406853	12:43040189	0.442	-0.655	1.03E-03
	rs7316154	12:43041140	0.442	-0.649	1.14E-03
	rs10880336	12:43042430	0.383	-0.657	1.14E-03
	rs34393555	12:43041863	0.442	-0.645	1.24E-03
